# A revision of the genus Amamiclytus Ohbayashi from Taiwan and the Ryukyu Islands (Coleoptera, Cerambycidae)

**DOI:** 10.3897/zookeys.118.1165

**Published:** 2011-07-13

**Authors:** Tatsuya Niisato, Chang-do Han

**Affiliations:** 1 Bioindicator Co., Ltd., Hara-machi 3–19, Shinjuku, Tokyo, 162–0053 Japan; 2 Entomological Laboratory, Faculty of Agriculture, Ehime University, Tarumi, Matsuyama, 790–8566 Japan

**Keywords:** *Amamiclytus*, Clytini, revision, new species, Taiwan, Ryukyu Islands

## Abstract

The clytine genus *Amamiclytus* Ohbayashi, 1964, from Taiwan and the Ryukyu Islands is revised. Seven species and one subspecies are recognized as follows: *Amamiclytus nobuoi nobuoi* Ohbayashi, 1964, (Amami Islands); *Amamiclytus nobuoi akusekianus* Niisato, 2005, (Tokara Islands); *Amamiclytus subnitidus* Holzschuh, 1984, (Taiwan); *Amamiclytus setiger* **sp. n.** (Taiwan), *Amamiclytus nubilus* **sp. n.** (Taiwan), *Amamiclytus juni* **sp. n.** (Taiwan), *Amamiclytus yulongi* **sp. n.** (Taiwan) and *Amamiclytus hirtipes* (Matsushita, 1940), **comb. n.** (Taiwan). All of them are described or redescribed and a key to species is presented. The generic features of *Amamiclytus*, including male and female genitalia from these taxa, are presented. The systematic position of *Amamiclytus* within the tribe Clytini is discussed.

## Introduction

Clytine beetles of the genus *Amamiclytus* Ohbayashi, 1964, are easily recognized at first observation by the small, black and glossy body with the ordinary arrangement of white pubescent maculation on the elytra. The genus is distinguishable from related genera, like *Rhaphuma* Pascoe, by rather widely separated antennal insertions, long, erect, pale hairs on the middle and hind tibiae, and also by the peculiar structure of the male endophallus. Up to the present time, a total of seven taxa, including one subspecies, have been recorded from a rather restricted area between the Ryukyu Islands of Southwest Japan and northern Indochina. They are *Amamiclytus nobuoi nobuoi* Ohbayashi, 1964, from Amami Isls., Japan, *Amamiclytus nobuoi akusekianus* Niisato, 2005, from Tokara Isls., Japan, *Amamiclytus hirtipes* (Matsushita, 1940), from Taiwan, *Amamiclytus subnitidus* Holzschuh, 1985, from Taiwan, *Amamiclytus dembickyi* Holzschuh, 1991, from North Vietnam, *Amamiclytus squamifer* Holzschuh, 1991, and *Amamiclytus setosulus* Holzschuh, 1991, from Northwest Thailand.

In spite of Taiwan’s rich longicorn beetles fauna, only two species of this genus, *Amamiclytus hirtipes* and *Amamiclytus subnitidus*, were known from there. The first author preliminarily examined the Taiwanese *Amamiclytus* species more than a quarter century ago, and presented an oral report at the annual meeting of the Japanese Society of Coleopterology (Niisato 1983). According to his provisional report, a total of six species in the genus were recognized from Taiwan, of which five were unnamed species.

In this study, we basically follow the work of Niisato (1983), and carefully describe or redescribe the members of the genus from Taiwan and the Ryukyu Islands considering details of external morphology and male and female genitalia. We also discuss the systematic position of *Amamiclytus* in the tribe Clytini.

### Abbreviations

**Measurements of body parts.** HW–width of head across eyes; PL– length of pronotum; PW– maximum width of pronotum; PA–apical width of pronotum; PB–basal width of pronotum; EL–length of elytra; EW–humeral width of elytra; M–arithmetic mean.

**Maculations consisting of white pubescence.** Dorsal side: Pb–basal band on pronotum, usually enlarged along entire margin; B–basal bands near elytral bases; S–sutural spot on elytra behind scutellum; La–lateral bands before middle of elytra, sometimes almost reaching or joining S; Lp–transverse bands behind middle of elytra, usually incomplete, not reaching external and sutural margins; A–apical bands of elytra. Ventral side: Msl–lateral maculation of mesosternum; Mss–maculation on mesosternal process; Mta–L-shaped band along apical margin of metathorax, extending to apical 1/2–2/3 of metepisternum; V1–V4–lateral bands along apical margins of ventrites 1–4, usually enlarged along entire apical margin in basal segments.

**Depositories of type specimens.** EUMJ–Ehime University Museum, Matsuyama, Japan; HUM–Hokkaido University Museum, Sapporo, Japan; MMNS–National Museum of Natural Science, Taichung, Taiwan; NHMO–Natural History Museum of Osaka, Japan; NMNS–National Museum of Nature and Science, Tokyo, Japan.

## Historical review

The genus *Amamiclytus* Ohbayashi, 1964, was established for *Amamiclytus nobuoi* Ohbayashi from Amami-Ôshima Is., Southwest Japan. Later, this type species was synonymized with the Taiwanese species, *Rhaphuma hirtipes* Matsushita, 1940, by Kojima et al. (1965). They also re-affirmed the validity of the genus *Amamiclytus* and placed it under *Amamiclytus* as *Amamiclytus hirtipes* (Matsushita, 1940). According to their paper, this action was made by comparison with specimens determined as *Rhaphuma hirtipes* by the late Dr. M. Hayashi from his collection. Later, Kusama and Takakuwa (1984) restored *Amamiclytus nobuoi* as a valid species. However, they never examined the true *Rhaphuma hirtipes*, and confused several different species in the genus with it. So at least as of that time, it was uncertain whether *Rhaphuma hirtipes* should be transferred to the genus *Amamiclytus* or not.

The holotype of *Rhaphuma hirtipes* was assumed to be deposited in an institution somewhere in Berlin, or lost during the war. However, the first author discovered the holotype of *Rhaphuma hirtipes* in the general collection of the Hokkaido University. By examination of this type specimen, Niisato (1983) accepted the placement in the genus *Amamiclytus*, and recognized six species in the genus, of which five needed to be described as new taxa for the Taiwanese fauna. After Niisato’s presentation, *Amamiclytus subnitidus* Holzschuh, 1984, was described from Kaohsiung, Taiwan. Until now, four taxa in this genus from Taiwan have remained undescribed.

Based on our recent examination of the late Dr. Masao Hayashi’s collection deposited in the Natural History Museum of Osaka, we only located a single species, *Amamiclytus subnitidus*, in his collection. This indicates that the previously incorrect action by Kojima et al. (1965) was due to the misidentification of *Amamiclytus subnitidus* with *Amamiclytus hirtipes*. Succeeding authors of the picture books of Taiwanese Cerambycidae (Yu and Nara 1988, Yu et al. 2002, Hua et al. 2009) inherited this mistake.

## Taxonomy

### Genus Amamiclytus

Ohbayashi, 1964

http://species-id.net/wiki/Amamiclytus

Amamiclytus Ohbayashi 1964: 21, pl. 4, fig. 1; type species: *Amamiclytus nobuoi* Ohbayashi, 1964.

#### Description.

Small to very small clytine with black, glossy body arranged with white pubescent maculations on pronotum, elytra and ventral surface, characterized by relatively well-separated eyes, and dense hair on middle and hind tibiae. Colour mostly black, glossy, sometimes more or less matted, usually with brownish antennae and legs, and sometimes also on meso- and metathoraces, and abdomen. Hairs and pubescence mostly very short though some partially dense and long; head usually sparsely clothed with pale gray pubescence on frons in both sexes, though more or less sparsely so in ♀; antennae with long, pale brown hairs along underside of 2nd to 4th or 5th segments; pronotum thinly pubescent, with white pubescent band along base (*Pb*), transverse or separated at sides, though sometimes sparse or quite absent according to species; scutellum with fine pale pubescence; elytra basically with three white maculations: 1) semicircular maculation or slightly oblique short band on basal third to fourth (*La*), 2) almost complete arcuate band on apical third to 2/5 (*Lp*), 3) narrow but usually clear band at apices (*A*), supplemented with the following maculation according to species: 4) basal narrow band (*B*), 5) sutural spot behind scutellum (*S*); prosternum with white pubescence on basal half to 2/3; metasternum with white pubescence at sides (*Msl*) and on intercoxal process (*Mss*); metathorax with L-shaped band along apical margin of metasternum, extending to apical half to 2/3 of metepisternum (*Mta*); abdomen with white pubescence at sides of ventrites 1–2, sometimes with same, though sparse maculation on ventrite 3 or ventrites 3–4 (*V1*–*V4*).

Head across eyes almost equal to the width of pronotum; frons almost quadrate, flattened, provided with a fine smooth median line, usually closely or coarselypunctured; clypeus flattened or slightly raised; mandible short and broad, rather strongly hooked near apex, with almost smooth inner margin, covered with numerous short hairs on surface, a few long hairs on outer margin near apex; maxilla with galea and lacinia weakly developed, terminal segment of palpus clearly flabellate, strongly dilated apicad in ♂, weakly so in ♀; terminal segment of labial palpus strongly dilated apicad in ♂, weakly so in ♀; vertex raised towards antennal cavities which are usually separated each other by half to 2/5 the width of occiput; occiput distinctly convex; genae relatively shallow, almost half to 1/3 the depth of lower eye-lobes in frontal view; eye moderately large, almost semicircular, a little narrower than frons in frontal view. Antennae thin, moderate in length or relatively long, slightly thickened apicad except for those of *Amamiclytus hirtipes* which are simply slender; scape almost cylindrical, 3rd segment 1.5–2.0 times as long as 4th segment, terminal segment usually obtusely pointed.

Pronotum globose or slightly elongate, slightly narrower than elytra, simply arcuate at sides; disc distinctly convex though slightly depressed above, provided with large shallow punctures. Scutellum small, regular triangular.

Elytra relatively short to relatively long; sides rounded at humeri, weakly arcuately emarginate at a level between basal fourth and apical half; apices oblique, slightly arcuate at margins, usually with weak dents at external angles; disc evenly convex with slight depression near suture just behind scutellum. Hind wing with vein Cu not attaining AA3+4 which is forming an ordinary H-shape.

Ventral surface smooth; prosternum moderately emarginate near apical half in profile; metasternum slightly convex; abdomen relatively slender, with male anal ventrite arcuately rounded at apical margin.

Legs long and slender; hind legs 1.5–2.0 times as long as elytra, with femur gradually swollen apicad, slightly compressed, usually exceeding elytral apex at apical fifth, almost equal to the length of hind tibia, 1st tarsal segment varied in length according to species, 1.5–2.5 times as long as the following two segments combined.

Male genitalia. Relatively large and somewhat elongate, basically related to that of several species of *Rhaphuma*. Median lobe elongate and slender, slightly reflexed in profile; dorsal plate almost equal in width to, or a little longer than ventral plate, gently narrowed to rounded apex; ventral plate gently narrowed to pointed apex; median struts slender. Endophallus about twice the length of median lobe, provided with minute or medium-sized spinous spicules behind crescent-like sclerites, densely covered with minute serrate or crenulate spicules on apical part. Tegmen usually elongate, shorter than median lobe; parameres nearly half to 2/5 the length of tegmen, divided in apical fifth to third, with each lobe almost rounded at apex, which is provided with short and long setae; basal ridge raised. Eighth tergite more or less elongate, slightly longer than wide. Eighth sternite distinctly transverse, emarginate or transversely truncate at apical margin, provided with long projection at middle of basal margin.

Female genitalia. Coxite lobe ovoid, scattered with short and long setae. Stylus half to equal in length to coxite lobe, elongate, weakly dilated apicad. Spermatheca narrow, weakly broadened apicad; gland short and thin; duct relatively long and thin.

#### Geographical distribution.

Indochina, China, Taiwan, Japan (Ryukyus), Malay Peninsula, Sumatra and Borneo.

#### Comments.

The species of the genus *Amamiclytus* Ohbayashi have so far been known from a rather restricted area between Indochina and the Ryukyu Islands of Southwest Japan. However, several undetermined species belonging to the genus have been found from Sumatra, Borneo and the Malay Peninsula. The genus contains small, glossy black species with pure white pubescent maculations on the body surface, and are slightly similar in external appearance to several members of the genus *Rhaphuma* Pascoe. They share the following features: 1) body relatively elongate, especially in elytra, meso- and metasterna, and legs; 2) antennae thin and long, with 3rd segment usually longer than scape; 3) antennal cavities approximate each other; 3) eyes large and prominent, rather distinctly approximate in front; 4) mandible almost smooth and provided with numerous short hairs along inner margin; 5) labial and maxillary palpi show distinct sexual dimorphism in each terminal segment, strongly dilated apicad in ♂ or weakly so in ♀. However, *Amamiclytus* is very distinct in the tribe Clytini by a combination of the following characters: 1) body small and rather convex, with relatively long antennae and legs; 2) colour wholly black, usually brownish antennae and legs, strongly glossy or sometimes more or less matted; 3) white scaly pubescence form the maculations on elytra, ventral surface and sometimes on the base of pronotum, of which elytra are always provided with three arcuate or transverse bands, sometimes supplemented with vague basal band along basal margin and a longitudinal spot near suture behind scutellum; 4) mid and hind tibiae provided with long erect hairs; 5) frons relatively wide, with a fine median groove; antennae usually thickened towards apical segments, though simply slender in *Amamiclytus hirtipes*, with antennal cavities widely separated at sides of frons; 6) pronotum rather convex, simply arcuate at sides; 7) male genitalia relatively large, with endophallus densely provided with minute serrate or crenulate spicules on apical part, without spinous spicules.

Adults of the genus are usually found on various types of tree blossoms as *Castanopsis*, *Quercus* and *Acer* mainly in spring and early summer season, such as February to June, except for *Amamiclytus hirtipes* which appears in the autumn season. Larvae of *Amamiclytus nobuoi*, bore in the dead, thin twigs of *Machilus thunbergii* and *Rhaphiolepis indica* var. *umbellata* (Niisato, 2007).

#### Key to species of the genus *Amamiclytus* from Taiwan and the Ryukyu Islands

**Table:** 

1	Pronotum longer than wide, weakly or moderately arcuate at sides, usually provided with white basal band	2
–	Pronotum as long as, or slightly longer, than wide, usually strongly arcuate at sides, without white pubescence near basal margin	6
2	Elytra glossy, sparsely clothed with very short pale pubescence	3
–	Elytra matted, densely clothed with brownish pubescence	5
3	Elytra provided with white spot near suture behind scutellum; strongly glossy, sparsely clothed with pale pubescence	*Amamiclytus nobuoi* Ohbayashi
–	Elytra without white spot near suture behind scutellum	4
4	Elytra moderately glossy, without long pale hairs; frons distinctly longer than wide; erect pale hairs of hind tibia sparse and not so long	*Amamiclytus subnitidus* Holzschuh
–	Elytra strongly glossy, scattered with a few erect long pale hairs; frons almost as long as wide; erect pale hairs of hind tibiae long and dense	*Amamiclytus setiger* sp. n.
5	Pronotum provided with distinct basal white band; elytra without white spot near suture behind scutellum	*Amamiclytus nubilus* sp. n.
–	Pronotum not forming a distinct basal white band, though sparsely clothed with white pubescence near basal margin; elytra with white spot near suture behind scutellum	*Amamiclytus hirtipes* (Matsushita), comb. n.
6	Pronotum finely punctured; elytra with white bands before middle arcuate, usually reaching to sutural white spot, white bands behind middle arcuate; abdomen without lateral white bands on ventrites 3–4	*Amamiclytus juni* sp. n.
–	Pronotum coarsely punctured; elytra with white bands before middle semicircular, usually isolated but rarely arcuate and almost reaching to sutural white spot, lateral bands behind middle almost transverse or slightly oblique; abdomen provided with lateral white bands on ventrites 3–4	*Amamiclytus yulongi* sp. n.

### Amamiclytus nobuoi nobuoi

Ohbayashi, 1964

http://species-id.net/wiki/Amamiclytus_nobuoi_nobuoi

Figs 3 6–7 34 41–46

Amamiclytus nobuoi Ohbayashi, 1964: 21, pl. 4, fig. 1; type locality: Hatsuno, Amami-Ôshima.
Amamiclytus hirtipes : Kojima et al. 1965: 85. (nec Matsushita 1940)
Amamiclytus nobuoi nobuoi : Niisato 2007: 502.

#### Description.

Male and female. Body length (from vertex to elytralapices) 4.0–5.1 mm in ♂, 4.0–4.9 mm in ♀. Colour black, glossy in general, distinctly so on elytra, dark brown in antennae, meso- and metathoraces, abdomen and legs except for pale brown tibiae and yellowish brown tarsi, mouthparts except for black mandibles yellowish brown. Body moderately clothed with fine pale pubescence, sparsely with erect long pale hairs on clypeus, genae, near pronotal base, abdominal ventrites and hind tibiae; head sparsely with pale hairs, largely exposing disc, scattered with a few erect long pale hairs, very thinly with pale gray pubescence on frons in both sexes; antennae distinctly with long pale brown hairs along undersides of segments 2–5; pronotum largely exposing disc, thinly pubescent, with a few erect long pale hairs, *Pb* at sides transverse, slightly narrowed inwards; scutellum hardly pubescent; elytra sparsely with pale pubescence, without long erect pale hairs, *B* very sparse, barely recognized, *S* on basal eighth small and longitudinally oblong, *La* on basal third semicircular, slightly oblique, *Lp* on apical third, almost complete though narrow, weakly arcuate, *A* moderately dense; prosternum moderately with white pubescence near basal 2/3, *Msl* distinct, *Mss* almost absent, *Mta* very sparse along posterior margin of metasternum, dense and entire on apical 2/5 of metepisternum; *V1* and *V2* dense, narrowed to middle.

Head across eyes almost as wide as pronotum, HW/PW 0.80–1.06 (M 0.95) in ♂, 0.89–0.95 (M 0.92) in ♀; frons as long as wide, arcuately dilated apicad, with a fine but deep smooth median line, closely roughly punctured; clypeus almost flattened; vertex raised towards antennal cavities which are separated from each other by about half the width of occiput; occiput distinctly convex, sparsely punctured. Antennae thin and long, gradually thickened apicad, attaining apical third in ♂ or 3/5 in ♀ of elytra; scape almost cylindrical, 3rd segment 1.4–1.8 times as long as 4th segment, middle segments weakly thickened at apices, terminal segment short, rounded at apex in ♂.

Pronotum slightly longer than wide, strongly arcuate at sides; PL/PW, 1.18–1.38 (M 1.27) in ♂, 1.09–1.20 (M 1.13) in ♀, PB/PA 0.92–1.00 (M 0.95) in ♂, 0.87–1.00 (M 0.92) in ♀, EL/PL 2.40–3.00 (M 2.68) in ♂, 2.67–3.11 (M 2.92) in ♀, PW/EW 0.76–0.91 (M 0.83) in ♂, 0.79–0.82 (M 0.80) in ♀; disc moderately convex though slightly depressed above, provided with large shallow punctures, though densely finely punctured in apical half in ♂. Scutellum regular triangular, slightly acute at apex.

Elytra relatively long and slender, EL/EW 2.73–2.86 (M 2.78) in ♂, 2.55–2.80 (M 2.64) in ♀; sides with almost rounded humeri, gently arcuate at a level between basal fourth and apical half, then gently arcuate and narrowed to oblique apices, without any dent at inner and outer angles; disc almost evenly convex, sparsely provided with fine shallow punctures.

Ventral surface almost smooth, provided with a few coarse punctures on prosternum, minute ones on meso- and metathoraces and abdomen; ♂ anal ventrite 3/5 the length of basal width, weakly arcuate at apical margin.

Legs slender, rather long; hind legs 1.4–1.7 times as long as elytra, with femur strongly swollen in apical half, slightly exceeding elytral apex, 1st tarsal segment 1.6–2.0 times as long as the following two segments combined.

Male genitalia. Median lobe about 2/5 the length of elytra, slender and elongate; dorsal plate slightly wider than ventral plate in apical eighth, distinctly narrowed to apex which is rounded; ventral plate almost parallel-sided in basal half, then strongly narrowed to apex which is strongly pointed at the extremity, slightly reflex in profile; median struts slender, almost half the length of median lobe. Endophallus densely provided with minute spinous spicules behind crescent-like sclerites at a level between basal 2/5 and 4/5, densely covered with minute serrate spicules on apical sixth. Tegmen elongate, slightly shorter than median lobe; parameres more or less elongate, nearly 2/5 the length of tegmen, divided in apical fourth, gently arcuate in external margins, arcuately emarginate in inner margins, almost rounded at apices which are provided with numerous short and a few long setae; basal ridge slightly raised; ring part almost parallel-sided in basal third. Eighth tergite slightly elongate, semicircular, gently narrowed apicad in apical fourth, with apical margin weakly arcuate, provided with numerous short and a few long setae. Eighth sternite very small, distinctly transverse, almost 1/3 the length of median lobe, with apical margin almost transverse, deeply concave at middle.

Female genitalia. Coxite lobe ovoid, scattered with short and long setae. Stylus almost half in length to coxite lobe, elongate, weakly dilated apicad. Bursa copulatrix small, weakly constricted near base. Spermatheca narrow, gently arcuate, weakly broadened apicad; gland thin, attached near apical third; duct thin, strongly coiled throughout.

#### Specimens examined.

1♀ (holotype), Hatsuno, Amami-Ôshima, 10–VII–1962, N. Ohbayashi leg. (EUMJ); 2♀♀, Hatsuno, Amami-Ôshima Is., Kagoshima Pref., 28–VI–1970, K. Sakai leg.; 1♂, 1♀, same locality, 24–VI–1970, T. Kobayashi leg.; 1♂, 1♀, same locality, 19–VI–1971, H. Yokoyama leg.; 1♂, same locality and collector, 29–VI–1971; 1♀, same locality and collector, 30–VI–1971; 1♀, same locality, 6–VII–1973, M. Ito leg.; 1♂, same locality, 6–VII–1975, K. Kuzugami leg.; 1♂, Chûô–Rindô, Naze City, Kagoshima Pref., 30–VI–2001, T. Kurihara leg.; 1♀, Ôganeku, Yamato Village, Ôshima Gun, Kagoshima Pref., 30–VI–2003, T. Kurihara leg.

#### Geographical distribution.

Amami Isls. (Amami-Ôshima Is.), Ryukyus, Japan.

#### Comments.

The nominotypical subspecies of *Amamiclytus nobuoi* Ohbayashi is endemic to the Amami-Ôshima Isls., mid-Ryukyus of Southwest Japan. The adult beetles mainly appear in summer between mid June and early July, and are often found on tree blossoms. The larvae bore in freshly dead twigs of *Rhaphiolepis indica* var. *umbellata* and *Machilus thunbergii* (Niisato, 2007). Though not so rare until 1970’s, only a few specimens of *Amamiclytus nobuoi nobuoi* have been collected since the 1980’s. This clytine has been recently treated as “Data Deficient”, a category of endangered animals in the Japanese Red Data Book (Ministry of the Environment, Japan, 2007).

This species is somewhat similar in external morphology to *Amamiclytus setiger* sp. n. from Taiwan, but can be easily distinguished from the latter by the elytra lacking erect long pale hairs and quite different structure of the male genitalia.

**Figures 1–2.**
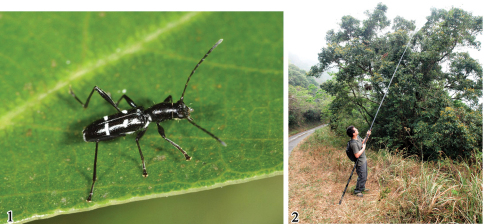
*Amamiclytus subnitidus* Holzschuh and its collecting site: **1** Male habitus **2** Mt. Dahan Shan, Pingdong County, S. Taiwan. Photo by Y. Ito on 4. April. 2010.

**Figures 3–5.**
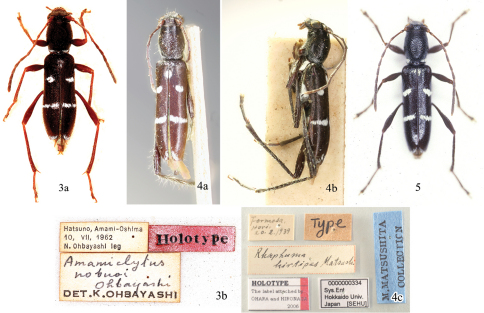
Holotypes of *Amamiclytus* spp.: **3** *Amamiclytus nobuoi* Ohbayashi (in EUM), a, habitus in dorsal view, b, labels; **4** *Rhaphuma hirtipes* Matsushita (in HUM), a, habitus in dorsal view, ditto in lateral view, c, labels; **5** *Amamiclytus subnitidus* Holzschuh (in Holzschuh coll.).

**Figures 6–14.**
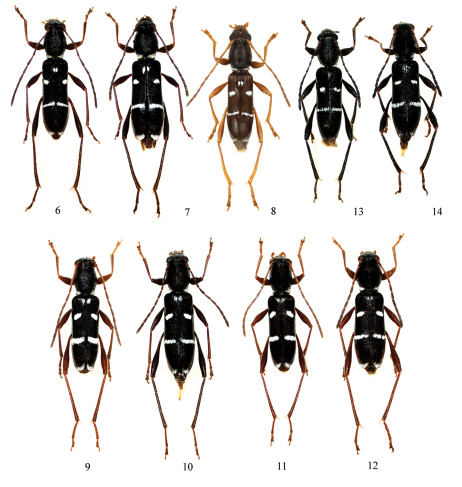
*Amamiclytus* spp. from Japan and Taiwan: **6** *Amamiclytus nobuoi nobuoi* Ohbayashi, ♂ **7** ditto, ♀ **8** *Amamiclytus nobuoi akusekianus* Niisato, holotype ♂ **9** *Amamiclytus subnitidus* Holzschuh, ♂ **10** ditto, ♀ **11** ditto (Bilyu, Xiulin Township), ♂ **12** ditto, ♀ **13** *Amamiclytus setiger* sp. n., holotype ♂ **14** ditto, allotype ♀.

### Amamiclytus nobuoi akusekianus

Niisato, 2005

http://species-id.net/wiki/Amamiclytus_nobuoi_akusekianus

Fig. 8

Amamiclytus nobuoi akusekianus Niisato 2005: 383, figs 1a, b, 2b, d, f; type locality: Akuseki-jima Is., Tokara Isls., N. Ryukyus, Kagoshima Pref., Japan.

#### Description.

Differentiated from the nominotypical subspecies from Amami-Ôshima Is. by the following features: 1) antennae and legs yellowish brown, instead of chocolate brown; 2) legs shorter, with hind femora not reaching elytral apices even in ♂; 3) tergite 8 gently arcuate at apical margin instead of transversely truncate; 4) median lobe longer and more slender, with apical part narrowed in straight line to the extremity and well exposed in ventral view.

#### Specimens examined.

1♂ (holotype) (NMNS), 1♀ (paratype), Akuseki-jima Is., Tokara Isls., N. Ryukyus, Kagoshima Pref., Japan, host collected on 16~17–II–1986, emerged out on 5–VII–1986, K. Mori leg.; 1♂ (paratype), same data as the preceding but on 12–VI–1986; 1♂ (paratype), same locality as the holotype, emerged on 5–VI–1987.

#### Geographical distribution.

Tokara Isls. (Akuseki-jima Is.), Ryukyus, Japan.

### Amamiclytus subnitidus

Holzschuh, 1984

http://species-id.net/wiki/Amamiclytus_subnitidus

Figs 1 5 9–12 26–33 35 47–52

Amamiclytus subnitidus Holzschuh, 1984: 358, fig. 6; type locality: Formosa, Takao Hsien, Laopi.
Amamiclytus hirtipes : Kojima et al. 1965: 74; Yu et al. 2001: 97, pl. 14, fig. 22; Chou 2004: 207.

#### Description.

Male and female. Body length (from vertex to elytral apices) 3.2–5.1 mm in ♂, 3.7–5.3 mm in ♀. Colour black, more or less glossy in general, distinctly so on elytra, dark brown on antennae, meso- and metathoraces, abdomen, brown on legs except for yellowish-brown tarsi, mouthparts, except for black mandibles, yellowish-brown. Body sparsely clothed with fine pale pubescence, with a few erect long pale hairs on genae, pronotum, pro- and mesosterna, abdominal ventrites, and along undersides of mid and hind legs except for tarsi; head sparsely clothed with pale hairs, and moderately with pale gray pubescence on frons and genae in ♂, sparsely so in ♀; antennae with long pale brown hairs along undersides of segments 2–4; pronotum thinly pubescent, sparsely with erect long pale hairs, *Pb* at sides transverse, slightly narrowed inwards; scutellum thinly with pale pubescence; elytra rather thinly with pale pubescence, without erect, long, pale hairs, *B* around scutellum rather densely pubescent, *S* absent, *La* on basal fourth semicircular, distinctly oblique, *Lp* on apical 2/5, almost complete, relatively narrow, slightly arcuate, *A* along apical margin densely pubescent; ventral surface with *Ps* dense near basal 2/3, *Msl* and *Mss* distinct, *Mta* dense along posterior margin of metasternum except near middle, distinctly dense and entire on apical 2/3 of metepisternum; *V1* and *V2* at sides dense, narrow to middle, *V3* feebly present at sides near apical margin, though sometimes disappeared.

Head across eyes almost as wide as pronotum, HW/PW 0.91–1.13 (M 0.99) in ♂, 0.83–1.00 (M 0.91) in ♀; frons distinctly wide, about 1.7 times as wide as long, arcuately dilated apicad, with a fine shallow smooth median line, closely roughly punctured; clypeus slightly convex; vertex raised towards antennal cavities which are separated each other by 2/5 the width of occiput; occiput distinctly convex, feebly sparsely punctured. Antennae thin and long, gradually thickened apicad, attaining apical fifth in ♂ or 2/5 in ♀ of elytra; 3rd segment 1.5–2.0 times as long as 4th segment, terminal segment elongate in ♂.

Pronotum rather distinctly longer than wide, completely arcuate at sides; PL/PW, 1.05–1.33 (M 1.21) in ♂, 1.00–1.33 (M 1.15) in ♀, PB/PA 0.82–1.08 (M 0.95) in ♂, 0.92–1.00 (M 0.95) in ♀, EL/PL 2.36–3.27 (M 2.80) in ♂, 2.67–3.30 (M 2.95) in ♀, PW/EW 0.76–0.95 (M 0.84) in ♂, 0.68–0.80 (M 0.74) in ♀; disc moderately convex though slightly depressed above, provided with large shallow punctures. Scutellum regularly triangular, slightly acute at apex.

Elytra moderate in length, rather slender, EL/EW 2.58–3.13 (M 2.83) in ♂, 2.27–2.70 (M 2.51) in ♀; sides rather strongly rounded at humeri, gently arcuate at a level between basal fourth and apical half, then gently arcuate and narrowed to apices which are oblique, without any dent at inner and outer angles; disc evenly convex, sparsely provided with deep, more or less coarse punctures.

Ventral surface almost smooth, provided with a few coarse punctures on prosternum, minute ones on meso- and metathoraces and abdomen; ♂ anal ventrite 3/10 the length of basal width, truncate at apical margin.

Legs slender, relatively long; hind legs 1.5–2.0 times as long as elytra, with femur strongly swollen in apical half, and exceeding elytral apices at apical fifth, 1st tarsal segment 1.7–2.4 times as long as the following two segments combined.

Male genitalia. Median lobe about 1/3 the length of elytra moderately elongate and slender; ventral plate almost equal in width to or slightly shorter than ventral plate, gently narrowed to apex which is rounded; ventral plate almost parallel-sided near basal 2/5 then strongly narrowed to apex which is distinctly, strongly pointed at the extremity, weakly reflex in profile; median struts long and slender, almost 7/10 the length of median lobe. Endophallus densely provided with minute and medium-sized spinous spicules behind crescent-like sclerites at a level between basal 3/10 and 3/5, densely covered with minute serrate spicules on apical fourth. Tegmen slightly elongate, distinctly shorter than median lobe; parameres wide, slightly elongate, nearly half the length of tegmen, divided in apical third, gently arcuate in external margins, arcuately emarginate in inner margins, almost rounded at apices which are provided with numerous short and long setae; basal ridge moderately raised; ring part gently narrowed to apex which is widely expanded. Eighth tergite slightly elongate, almost quadrate, slightly dilated apicad in apical 2/5, apical margin weakly emarginate, provided with numerous short and a few long setae. Eighth sternite transverse, about 1/4 the length of median lobe, slightly emarginate at apical margin, strongly prominent at the middle of basal margin.

Female genitalia. Coxite lobe ovoid, scattered with four or five short setae. Stylus almost equal in length to coxite lobe, more or less elongate, gently dilated apicad. Bursa copulatrix large, distinctly constricted in basal half. Spermatheca, narrow, weakly arcuate; gland attached just behind apex; duct thin and strongly coiled throughout.

#### Specimens examined.

1♂ (holotype), Formosa, Takao Hsien, Laopi, 6–IV–1978, Kezuka leg. (Holzschuh coll.).  [Taoyuan County, N. Taiwan] 2♂♂, 1♀, near Mt. Lala Shan, Fuxing Township, 12–V–1978, T. Shimomura leg.; 1♂, same locality, 28–VI–1979, J.-J. Luo leg.; 2♂♂, Sihleng, Fuxing Township, 26–IV–1982, N. Ohbayashi leg. [Hsinchu County, N. Taiwan] 1♂, Dalu Forest Road, Wufeng Township, alt. 1,400m, 5–IV–1994, C.-C. Chen leg.; 2♂♂, 2♀♀, same locality and collector, 24–IV–1994; 2♂♂, same locality, alt. 1,100~1,400m, 18–V–2002, Y.-L. Lin leg.; 2♀♀, same locality and collector, 7–VII–2004; 1♂, 1♀, same locality and collector, 24–V–2009; 1♂, 1♀, same locality, East Feeder, 27~28–VI–2003, D.-I. Hwang leg.; 4♂♂, 1♀, Mt. Lidong Shan, Jianshi Township, alt. 1,400m, 22–V–2005, Y.-L. Lin leg.; 1♀, Yufong, Jianshi Township, alt. 800m, 4–IV–2009, Y.-L. Lin leg. [Taichung County, C. Taiwan] 2♂♂, Deji Reservoir, Heping Township, 30–IV–1982, N. Ohbayashi leg.; 3♂♂, 2♀♀, Mt. Anma Shan, Heping Township, 5~28–VI–2002, N. Ohbayashi leg.; 1♂, 1♀, Siangyang~Liyuan, alt. 1,793~2,250m, 8–VII–2005, S.-T. Hisamatsu leg.; 1♀, Lilengsi Forest Road, Heping Township, alt. 1,700m, 25–IV–2000, W.-I Chou leg. [Nantou County, C. Taiwan] 1♀, Cueifong, Ren’ai Township, 5–VI–1976, T. Matsumoto leg.; 1♂, Meifong, Ren’ai Township, 7–V–1973, K. Matsuda leg.; 1♂, 1♀, same locality, 27–IV–1977, S. Saito leg.; 1♀, Songgang~Meifong, Ren’ai Township, 3–V–1977, J. Ito leg.; 1♂, Meiyuan, Ren’ai Township, 20–IV–1995, M. Hayashi leg.; 3♂♂, same locality, 28–IV–1995, M. Yagi leg.; 1♀, Nanshansi, Ren’ai Township, 17–IV–1977, W. Suzuki leg.; 1♀, same locality, 13–IV–1978, M. Ito leg.; 1♂, same locality, 24–III–1979, T. Ito leg.; 1♂, same locality and collector, 21–IV–1982; 1♀, same locality, 31–III–1980, M. Yagi leg.; 1♂, 1♀, same locality, 28~30–III–1981, H. Torigai leg.; 1♂, 1♀, Lianhuachih, Yuchi Township, 16–III–1978, J. Ito leg.; 1♀, same locality, 25–III–1980, K. Matsuda leg.; 13♂♂, 6♀♀, same as the preceding but 27–III–1980; 1♀, Sun moon Lake, Yuchi Township, 9–IV–1978, M. Ito leg.; 1♂, same locality, 13–V–1996, C.-K. Yu leg.; 1♀, same locality, alt. 800m, 3–IV–2000, Y.-L. Lin leg.; 2♂♂, 1♀, Bilyusi, Ren’ai Township, alt. 2,200m, 12–V–2002, Y.-L. Lin leg.; 1♂, same locality, 15–V–2007, C.-C. Chen leg.; 3♂♂, Mt. Guandao Shan, alt. 1,500m, Ren’ai Township, 12–V–1984, J.-J. Luo leg.; 1♀, same locality, 23–V–1985, M. Yagi leg.; 1♀, same locality and collector, 20–IV–1986; 1♀, same locality, 5–V–1985, K. Kusama leg.; 2♂♂, same locality, 12–V–2007, Y.-L. Lin leg.; 2♂♂, 1♀, Gaofeng, Ren’ai Township, 22–III–2007, native collector leg.; 2♂♂, 1♀, same locality, 5–V–2009, J.-J. Luo leg. [Hualien County, E. C. Taiwan] 1♀, Bilyu, Xiulin Township, 8–V–1977, S. Saito leg.; 1♂, 1♀, same locality, 2–VI–1978, M. Ito leg.; 2♂♂, 1♀, same locality and collector, 9–VI–1978; 3♂♂, same locality and collector, 13–VI–1978; 3♂♂, same locality and collector, 14–VI–1978; 9♂♂, 1♀, same locality and collector, 15–VI–1978; 1♂, same locality and collector, 17–V–1978. [Chiayi County, S. Taiwan] 1♀, Jhaoping, Alishan Township, 9–VI–1938, Y. Yano leg. [Kaohsiung County, S. Taiwan] 1♀, Shanping, Maolin Township, 30–III–1978, K. Matsuda leg.; 1♂, Mt. Nanfenshan, Liouguei Township, 22–IV–1985, W.-R. Chen leg.; 2♂♂, 1♀, Tengjhih, Taoyuan Township, alt. 1,400m, 28–III–2007, Y.-L. Lin leg. [Pingdong County, S. Taiwan] 1♂, Siaoguei Lake, Wutai Township, alt. 1,500m, 2–V–1998, C.-C. Chen leg.; 1♂, 1♀, Mt. Dahan Shan, Chunri Township, alt. 1,200m, 21–IV–2005, Y.-L. Lin leg.; 1♂, same locality and collector, 13–IV–2009; 1♂, 1♀, same locality and collector, 5–V–2009.; 1♀, same locality, 16–IV–2007, T. Niisato leg.; 1♂, same locality and collector, 5–V–2009; 7♂♂, 1♀, same locality and collector, 3–V–2009. 1♂, Tiantizo, 1–VIII–1976, K. Matsuda leg.

#### Geographical distribution.

Taiwan.

#### Comments.

*Amamiclytus subnitidus* Holzschuh is closely related in external morphology and pubescent maculation to *Amamiclytus setiger* sp. n., but is clearly separable from the latter by the sparsely pubescent pronotum, the absence of erect long pale hairs on elytra, and the transverse frons which is 1.7 times as wide as long instead of being as wide as long. These two species are also easily distinguished by the male genitalia. This species has the elongate median struts which are about 7/10 the length of the median lobe, and has a rather strongly expanded base of the ring part of the tegmen.

The population from Bilyu, Xiulin Township in east-central part of the central mountains of Taiwan, shows a slight colour variation in antennae and legs which are clearly pale brown, but other characteristics agree quite well with the populations from other localities.

This is the most common species from the genus in Taiwan, and widely recorded from various localities north to south on the island. The adult beetles appear from early spring to early summer (March to June), and visit various tree blossoms.

**Figures 15–23.**
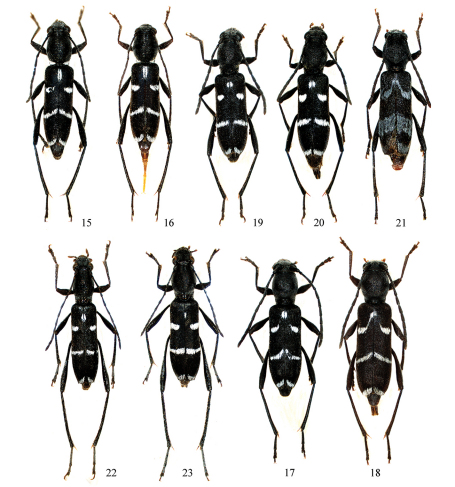
*Amamiclytus* spp. from Taiwan: **15** *Amamiclytus nubilus* sp. n., holotype ♂ **16** ditto, allotype ♀ **17** *Amamiclytus juni* sp. n., holotype ♂ **18** ditto, allotype ♀ **19** *Amamiclytus yulongi* sp. n., holotype ♂ **20** ditto, allotype ♀ **21** ditto, paratype ♂ **22** *Amamiclytus hirtipes* (Matsushita), comb. n., ♂ **23** ditto, ♀.

**Figures 24–33.**
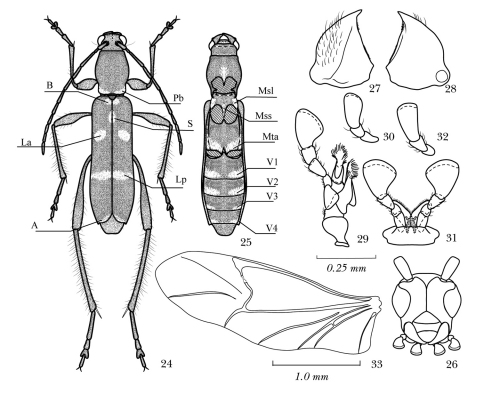
Body parts of *Amamiclytus* spp.: **24** Dorsal habitus showing white pubescent maculation (alphabetical codes see “Abbreviations”) **25** ditto, ventral view (V3 and V4 are hypothetically shown and absent in true *Amamiclytus hirtipes*) **26** head, frontal view **27** left mandibles, ventral view **28** ditto, ventral view **29** left maxilla, ventral view **30** ditto, ♀ **31** labium, ventral view **32** ditto, ♀ **33** left hind wing **24–25** *Amamiclytus hirtipes* (Matsushita), comb. n. **26–33** *Amamiclytus subnitidus* Holzschuh.

**Figures 34–40.**
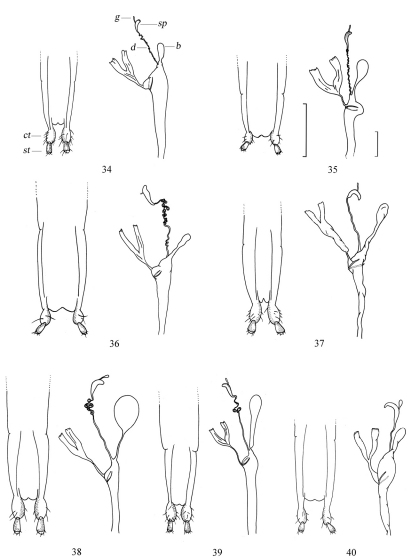
Female genitalia of *Amamiclytus* spp.: **34** *Amamiclytus nobuoi nobuoi* Ohbayashi **35** *Amamiclytus subnitidus* Holzschuh **36** *Amamiclytus setiger* sp. n. **37** *Amamiclytus nubilus* sp. n. **38** *Amamiclytus juni* sp. n. **39** *Amamiclytus yulongi* sp. n. **40** *Amamiclytus hirtipes* (Matsushita). ct coxite lobe st stylus b bursa copulatrix sp spermatheca d spermathecal duct g spermathecal gland. Scale: 0.5 mm.

### Amamiclytus setiger sp. n.

urn:lsid:zoobank.org:act:6ADAC35E-EE09-4256-8266-11B65684FB54

http://species-id.net/wiki/Amamiclytus_setiger

Figs 13–14 36 53–58 

#### Description.

Male and female. Body length (from vertex to elytral apices) 3.3–4.4 mm in ♂, 3.6–4.2 mm in ♀. Colour nearly same as *Amamiclytus  subnitidus*, though strongly glossy. Hairs almost as in *Amamiclytus subnitidus*, though partly, sparsely provided with erect long pale hairs, especially on elytra and hind tibia; head sparsely with pale hairs, very thinly with pale gray pubescence on frons in both sexes; *Pb* as in *Amamiclytus subnitidus*, though sparser; scutellum completely bare; elytra thinly with pale short pubescence, provided with a few erect very long pale hairs, with maculation almost as in *Amamiclytus subnitidus*, though *B* and *Lp* more sparse; venter of thoraces basically similar to that of *Amamiclytus subnitidus*, though *Ps* more sparse near basal half, *Mta* dense and entire on apical third of metepisternum; *V1*–*V3* almost as in *Amamiclytus subnitidus*; hind tibia densely with erect long pale hairs.

Head similar to *Amamiclytus subnitidus*, though frons almost as wide as long, slightly convex, with a weaker median line, clypeus almost flattened; HW/PW 0.93–1.08 (M 0.99) in ♂, 0.80–0.94 (M 0.89) in ♀. Antennae similar to *Amamiclytus subnitidus*, attaining apical 2/5 in ♂ or half in ♀ of elytra; 3rd segment 1.3–1.8 times as long as 4th segment, terminal segment more or less elongate in ♂. Pronotum almost as in *Amamiclytus subnitidus*, rather strongly arcuate near middle of sides; PL/PW 1.07–1.38 (M 1.25) in ♂, 1.06–1.13 (M 1.10) in ♀, PB/PA 0.92–1.00 (M 0.99) in ♂, 0.92–0.93 (M 0.93) in ♀, EL/PL 2.36–3.29 (M 2.75) in ♂, 2.44–2.89 (M 2.71) in ♀, PW/EW 0.80–0.88 (M 0.83) in ♂, 0.71–0.95 (M 0.86) in ♀; disc distinctly convex though depressed above, provided with large shallow punctures. Scutellum as in *Amamiclytus subnitidus*. Elytra almost as in *Amamiclytus subnitidus*, though rather slender, scattered with a few medium-sized punctures, with apices weakly acute at external angles; EL/EW 2.60–3.06 (M 2.85) in ♂, 1.83–2.94 (M 2.59) in ♀. Ventral surface almost as in *Amamiclytus subnitidus*, though sparsely punctured on abdomen; anal ventrite 2/5 the length of basal width in ♂, slightly arcuate at apical margin. Legs similar to *A*. *subnitidus*, though femur more strongly swollen in apical half, with 1st tarsal segment 1.7–2.3 times as long as the following two segments combined.

Male genitalia. Basically similar to that of *Amamiclytus subnitidus*, though median lobe larger and more slender, nearly 3/5 the length of elytra. Median lobe moderately elongate; dorsal plate almost equal in width to or a little shorter than ventral plate, distinctly narrowed to apex which is bluntly prominent; ventral plate almost parallel-sided near basal sixth then gently narrowing to apex, and strongly narrowed to near apical fourth, which is very prominent at the extremity, weakly reflexed in profile; median struts long and slender, almost 3/5 the length of median lobe. Endophallus densely provided with medium-sized spinous spicules behind crescent-like sclerites at a level between basal 3/10 and 3/5, densely covered with minute notched spicules on apical third. Tegmen more or less elongate, distinctly shorter than median lobe; parameres moderately wide, slightly elongate, almost half the length of tegmen, divided in apical third, gently arcuate in external margins, arcuately emarginate in inner margins, almost rounded at apices which are provided with short and a few long setae; basal ridge moderately raised; ring part almost parallel in basal 2/5. Eighth tergite almost quadrate, gently narrowed to apex in apical 3/5, almost transverse at apical margin, provided with numerous short setae. Eighth sternite transverse, almost 1/5 the length of median lobe, distinctly emarginate at apical margin.

Female genitalia. Almost as in *Amamiclytus subnitidus*, though bursa copulatrix weakly constricted in basal half, spermathecal duct more strongly coiled in apical half.

#### Type series.

Holotype ♂, Dalu Forest Road, Wufeng Township, alt. 1,400m, Hsinchu County, N. Taiwan, 5–IV–1994, C.-C. Chen leg. Allotype ♀, Yufong, Jianshi Township, alt. 800m, Hsinchu County, N. Taiwan, 19–IV–2002, Y.-L. Lin leg. Paratypes (17♂♂, 8♀♀): [Taoyuan County, N. Taiwan] 1♀, Shan-Paling, Fuxing Township, 24–V–1988, native collector leg. [Hsinchu County, N. Taiwan] 1♂, same data as the allotype; 1♂, same data as the holotype; 1♂, same locality, alt. 1,100~1,400m, 27–VII–2004, Y.-L. Lin leg. [Taichung County, C. Taiwan] 1♀, “Mt. DaKeng, Beitun District”, 16–V–1941, K. Seki leg. [Nantou County, C. Taiwan] 5♂♂, 3♀♀, Gaofeng, alt. 1,300m, Ren’ai Township, 8–VII–2007, N. Ohbayashi leg.; 2♂♂, Tseifong, Ren’ai Township, 1–V–1981, K. Kinugasa leg.; 1♂, Mt. Guandao Shan, Ren’ai Township, alt. 1,500m, 5–V–1985, K. Kusama leg.; 1♀, same locality, 6–VI–1995, S. Tsuyuki leg.; 1♂, 1♀, Nanshanshi, Ren’ai Township, 15–IV–1972, K. Matsuda leg. [Kaohsiung County, S. Taiwan] 1♂, Fengshan, 23–V–1977, K. Ushizima leg; 1♂, same locality and date, W.-L. Chen leg. [Formosa] 2♂♂, 1967, no further data; 1♀, 1967, no further data; 1♂, 1969, no further data. Holotype and allotype are preserved in NMNS, and paratypes are in EUMJ, HUM, MMNS, NHMO and the private collections of the above collectors.

#### Geographical distribution.

Taiwan.

#### Comments.

*Amamiclytus setiger* sp. n. is a distinctive species in having a very glossy body with erect, long, pale hairs especially on the elytral surface, and easily distinguished from the other members of the Taiwanese *Amamiclytus*. Concerning the morphology of male genitalia, this new species and *Amamiclytus subnitidus* Holzschuhshare several structures such as the distinctly long median struts, the ratio of the length between parameres and tegmen which is nearly half in length, similar forms of abdominal segment 8, and the similar pattern of spinous spicules behind crescent-like spicules on the endophallus. In female genitalia, the two species also have similar structure as shown in the above description. Therefore, these two species seem to form a species-group among the Taiwanese members of the genus.

This new species mainly appears in the summer season since most of the type series were collected in July, except for several paratypes collected in April and May.

**Figures 41–46.**
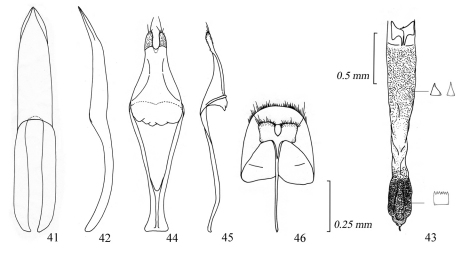
Male genitalia of *Amamiclytus nobuoi nobuoi* Ohbayashi: **41** Median lobe, ventral view **42** ditto, lateral view **43** endophallus **44** tegmen, ventral view **45** ditto, lateral view **46** abdominal segment 8, ventral view.

**Figures 47–52.**
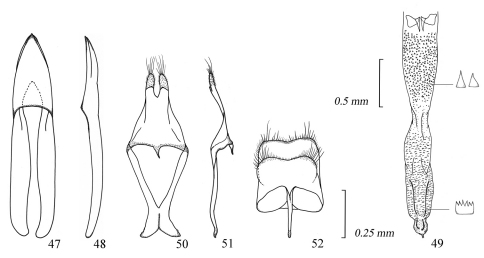
Male genitalia of *Amamiclytus subnitidus* Holzschuh: **47** Median lobe, ventral view **48** ditto, lateral view **49** endophallus **50** tegmen, ventral view **51** ditto, lateral view **52** abdominal segment 8, ventral view.

### Amamiclytus nubilus sp. n.

urn:lsid:zoobank.org:act:2AFA6B4C-76CA-4AA1-A054-BE3DFFB395CF

http://species-id.net/wiki/Amamiclytus_nubilus

Figs 15–16 37 59–64 

#### Description.

Male and female. Body length (from vertex to elytral apices) 3.6–4.5 mm in ♂, 4.0–5.2 mm in ♀. Colour almost as in *Amamiclytus subnitidus*, though distinctly matted in general. Body densely clothed with fine pale pubescence, partly with erect, long, pale hairs especially dense on mid and hind tibiae; head moderately with pale hairs, rather densely with pale gray pubescence on frons in ♂, sparsely in ♀; antennae moderately with pale pubescence, with long pale brown hairs on undersides of segments 2–5; pronotum rather densely with pale pubescence, with erect, long, pale hairs, *Pb* conspicuous; scutellum rather thinly with pale pubescence; elytra densely with light brown pubescence throughout, without erect, long, pale hairs, *B* absent, *S* on basal tenth dense and elongate, *La* on basal third strongly arcuate, slightly oblique, *Lp* on apical 2/5 almost complete, narrow, distinctly oblique, *A* distinct though rather narrow; prosternum densely with white pubescence near basal 2/3, *Msl* and *Mss* distinct, *Mta* dense and enlarged on entire of metepisternum; *V1*–*V4* dense, narrow to middle, though *V3*–*V4* sometimes sparse; fore and mid legs with pale gray pubescence, especially dense on femora.

Head almost as wide as pronotum, HW/PW 0.89–1.00 (M 0.99) in ♂, 0.89–1.00 (M 0.95) in ♀; frons as long as wide, arcuately dilated apicad, with a thin weak smooth median line, finely sparsely punctured; clypeus slightly convex; vertex raised towards antennal cavities which are separated from each other by half the width of occiput; occiput distinctly convex, sparsely punctured. Antennae thin and long, gradually dilated apicad, attaining apical half in ♂ or 3/5 in ♀ of elytra; 3rd segment 1.3–2.0 times as long as 4th segment, terminal segment short, rounded at apex in ♂.

Pronotum slightly or moderately longer than wide, weakly arcuate at sides; PL/PW, 1.13–1.38 (M 1.25) in ♂, 1.10–1.29 (M 1.19) in ♀, PB/PA 1.00–1.00 (M 1.00) in ♂, 1.00–1.06 (M 1.01) in ♀, EL/PL 2.45–3.22 (M 2.80) in ♂, 2.69–3.00 (M 2.86) in ♀, PW/EW 0.75–0.90 (M 0.84) in ♂, 0.75–0.83 (M 0.79) in ♀; disc moderately convex, provided with large shallow punctures. Scutellum regular triangular, acute at apex.

Elytra relatively long and slender; EL/EW 2.70–3.18 (M 2.94) in ♂, 2.50–2.83 (M 2.68) in ♀; sides distinctly rounded at humeri, gently arcuate at a level between basal fifth and apical half, then gently arcuate and narrowed to apices which are oblique, slightly arcuate at margins, minutely acute at outer angles; disc evenly convex, densely provided with fine shallow punctures.

Ventral surface almost smooth, provided with a few coarse punctures on prosternum, with minute punctures on meso- and metathoraces, and abdomen; anal ventrite in ♂, 3/5 the length of basal width, moderately arcuate at apical margin.

Legs slender, relatively long; hind legs 1.5–1.8 times length of elytra, with femur weakly swollen in apical half, exceeding elytral apices at apical fifth, 1st tarsal segment 1.5–2.0 times as long as the following two segments combined.

Male genitalia. Median lobe nearly 2/5 the length of elytra, wide, moderately elongate; ventral plate almost equal in width to, or a little shorter than ventral plate, gently narrowed to apex which is bluntly rounded; ventral plate almost parallel–sided near basal half then more or less strongly narrowed to apex which is distinctly pointed at the extremity, weakly reflexed in profile; median struts elongate, almost half the length of median lobe. Endophallus densely provided with minute spinous spicules behind crescent-like sclerites from basal 1/5 to 3/5, densely covered with minute serrate sclerites on apical third. Tegmen more or less elongate, distinctly shorter than median lobe; parameres elongate, slightly slender, nearly 2/5 the length of tegmen, divided in apical third, with lobe gently arcuate in external margin, arcuately emarginate in inner margin, almost rounded at apices which are provided with numerous short and a few long setae; basal ridge moderately raised, gently convergent to apex. Eighth tergite slightly elongate, semicircular, gently convergent to apex in apical half, with apical margin weakly arcuate, provided with numerous short setae. Eighth sternite more or less small, distinctly transverse, gently emarginate at apical margin.

Female genitalia. Coxite lobe ovoid, sparsely provided with short and long setae. Stylus almost half oflength ofcoxite lobe, elongate, gently dilated apicad. Bursa copulatrix small, strongly constricted in basal 2/3. Spermatheca slightly narrow, strongly arcuate; gland thin, attached at apical 2/5; duct more or less thin, slightly sinuate, not coiled.

#### Type series.

Holotype ♂, Sinsian, Wulai Township, Taipei County, alt. 300m, N. Taiwan, 4–III–2000, Y.-L. Lin leg. Allotype ♀, Lianhuachih, Yuchi Township, Nantou County, C. Taiwan, 20–III–1978, J. Ito leg. Paratypes (54♂♂, 23♀♀): [Taipei County, N. Taiwan] 1♂, Wulai, 21–II–2004, Y.-L. Lin leg.; 1♀, Sinsian, Wulai Township, alt. 300m, 11–III–2000, Y.-L. Lin leg.; 1♂, Fushan, Wulai Township, 21–III–1998, M. Sakai leg. [Taoyuan County, N. Taiwan] 3♂♂, Sule, Fuxing Township, 26–III–1979, T. Ito leg.; 1♂, same locality and collector, 28–III–1979; 6♂♂, 1♀, Sihleng, Fuxing Township, 26–IV–1982, N. Ohbayashi leg.; 1♂, same locality and collector, 28–IV–1982; 1♀, Baling, Fuxing Township, 31–III–1998, M. Sakai leg.; 1♀, Ronghua, Fuxing Township, 15–III–1998, C.-C. Chen leg.; 3♂♂, same locality, alt. 500m, 3–III–2002, Y.-L. Lin leg.; 6♂♂, Ronghua, Fuxing Township, alt. 500m, 27–III–2003, Y.-L. Lin leg.; 3♂♂, 1♀, Gaoyi, Fuxing Township, alt. 500m, 21–IV–2007, Y.-L. Lin leg. [Hsinchu County, N. Taiwan] 1♀, Dalu Forest Road, Wufeng Township, 17–IV–1994, C.-C. Chen leg.; 1♂, 1♀, same locality, alt. 1,100~1,400m, 24–III–2007, Y.-L. Lin leg.; 1♂, same locality and collector, 15–III–2009; 1♀, same locality and collector, 1–IV–2009.; 1♂, 1♀, Shihlei, Jianshi Township, 6–III–2010, Y.-L. Lin leg. [Nantou County, C. Taiwan] 10♂♂, 2♀♀, Lianhuachih, Yuchi Township, 16–III–1978, J. Ito leg.; 5♂♂, 1♀, same locality and collector, 17–III–1978; 1♂, same locality and collector, 18–III–1978; 1♀, Gaofeng, Ren’ai Township, alt. 1,300m, 5–V–2009, J.-J. Luo leg.; 1♀, Nanshansi, Ren’ai Township, 30–III–1972, K. Matsuda leg.; 1♂, same locality, 19–III–1978, J. Ito leg.; 1♂, same locality and collector, 22–III–1978; 1♀, same locality and collector, 26–III–1978; 1♂, 1♀, same locality, 11–IV–1978, M. Ito leg.; 1♂, 1♀, same locality and collector, 14–IV–1978; 1♂, same locality and collector, 29–IV–1978; 1♂, 1♀, same locality, 19–III–1978, C.-K. Yu leg.; 1♂, same locality, 17–III–1979, T. Ito leg.; 2♀♀, same locality and collector, 24–III–1979; 1♀, same locality, 28–III–1980, M. Yagi leg.; 1♀, same locality, 3–IV–1981, Y. Yamamoto leg. [Hualien County, E. C. Taiwan] 1♂, Bilyu, Xiulin Township, 2–VI–1978, M. Ito leg. [Kaohsiung County, S. Taiwan] 1♂, 8–III–1978, J. Ito leg.; 1♀, same collector, Wetuan, Liugui Township, 7–III–1978. [Pingdong County, S. Taiwan] 1♂, Mt. Dahan Shan, Chunri Township, 1–III–2007, T. Yoro leg. Holotype and allotype are preserved in NMNS, and paratypes are in EUMJ, HUM, MMNS, NHMO and the private collections of the above collectors.

#### Geographical distribution.

Taiwan.

#### Comments.

In having the matted body with similar pattern of white pubescent maculation on the elytra, *Amamiclytus nubilus* sp. n. is somewhat similar to *Amamiclytus juni* and *Amamiclytus yulongi* spp. n. which will be subsequently described, but is clearly distinguished from these two congeners by the relatively elongate pronotum with white pubescent maculation along the basal margin. Considering the male genitalia, this new species has some relationship with *Amamiclytus subnitidus* Holzschuh and *Amamiclytus setiger* sp. n., but is clearly separable from them by markedly broadened median lobe and rather narrow ring parts of tegmen. Besides, it is very unique that this new species and *Amamiclytus hirtipes* (Matsushita), comb. n. share an uncoiled duct in spermatheca of female genitalia in spite of strongly coiled ones in the other five species from Japan and Taiwan. The true affinity of *Amamiclytus nubilus* is uncertain since the morphological convergence is recognized in both external and genitalic features between some sympatric species of the genus. It may be possible to consider that *Amamiclytus nubilus* sp. n. has the closest relationship with *Amamiclytus hirtipes* (Matsushita), comb. n. by their uncoiled duct of spermatheca.

*Amamiclytus nubilus* sp. n. is widespread over the entire island of Taiwan and is rather common among the Taiwanese members of the genus. Adult beetles are usually found on various kinds of tree blossoms in the spring season mainly from March to April.

**Figures 53–58.**
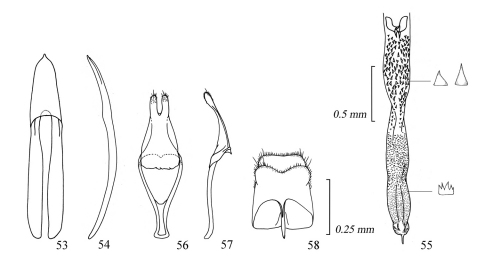
Male genitalia of *Amamiclytus setiger* sp. n.: **53** Median lobe, ventral view **54** ditto, lateral view **55** endophallus **56** tegmen, ventral view **57** ditto, lateral view **58** abdominal segment 8, ventral view.

**Figures 59–64.**
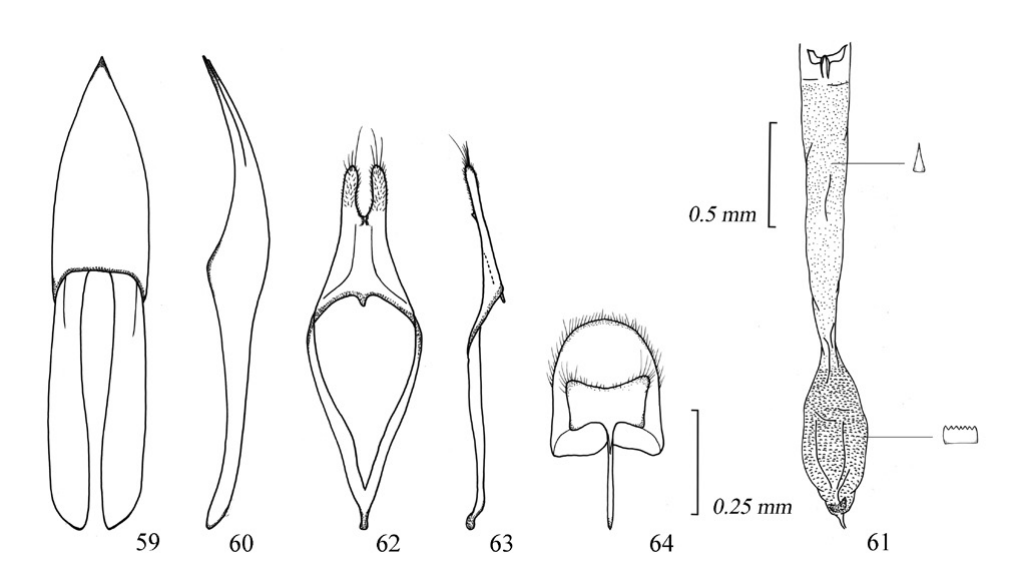
Male genitalia of *Amamiclytus nubilus* sp. n.: **59** Median lobe, ventral view **60** ditto, lateral view **61** endophallus **62** tegmen, ventral view **63** ditto, lateral view **64** abdominal segment 8, ventral view.

### Amamiclytus juni sp. n.

urn:lsid:zoobank.org:act:8260813A-D8BC-44E9-92E6-3BC303BFEF95

http://species-id.net/wiki/Amamiclytus_juni

Figs 17–18 38 65–70 

#### Description.

Male and female. Body length (from vertex to elytral apices) 3.40–4.70 mm in ♂, 3.70–5.20 mm in ♀. Colour black, more or less matted in general, dark brown on antennae, meso- and metathoraces, abdomen and legs, yellowish brown on mouthparts except for black mandibles. Body densely clothed with fine pale pubescence especially on pronotum and ventral surface, sparsely with erect long pale hairs on clypeus, genae, pronotal base, prosternal process and abdominal ventrites, mid and hind femora; head sparsely with pale gray pubescence on frons in ♂, sparsely so on that of ♀; antennae with long pale brown hairs along undersides of segments 2–4; pronotum densely with pale gray pubescence except for middle of disc forming a vague oblong black spot, *Pb* absent; scutellum thinly with pale pubescence; elytra densely with light brown pubescence throughout, *B* absent, *S* on basal eighth longitudinally oblong, *La* on basal 2/5 obliquely arcuate, usually attaining *S*, *Lp* on apical 2/5 almost complete, narrow, slightly arcuate, *A* rather narrow; *Ps* sparse near basal 2/3, *Msl* distinct, *Mss* completely absent, *Mta* rather sparse along posterior margin of metasternum, dense and entire on apical half of metepisternum; *V1* widely separated at sides, dense though narrowed inwards, *V2* feeble and sometimes disappeared.

Head across eyes almost as wide as pronotum, HW/PW 0.81–0.94 (M 0.88) in ♂, 0.80–1.00 (M 0.89) in ♀; frons as long as wide, arcuately dilated apicad, with a thin feeble smooth median line, densely, finely punctured; clypeus slightly convex; vertex raised towards antennal cavities which are separated from each other by half the width of occiput; occiput distinctly convex, rather densely coarsely punctured. Antennae thin and relatively short, gradually thickened apicad, attaining apical 2/5 in ♂ or half in ♀ of elytra; 3rd segment 1.4–2.0 times as long as 4th segment, terminal segment rather short and rounded at apex in ♂.

Pronotum almost equal in length to the maximum width near middle, strongly arcuate at sides; PL/PW 1.00–1.20 (M 1.08) in ♂, 0.92–1.29 (M 1.05) in ♀, PB/PA 0.81–0.93 (M 0.88) in ♂, 0.81–1.18 (M 0.94) in ♀, EL/PL 2.64–3.00 (M 2.92) in ♂, 2.72–3.22 (M 2.97) in ♀, PW/EW 0.71–0.83 (M 0.78) in ♂, 0.68–0.85 (M 0.78) in ♀; disc slightly convex, almost flattened above, densely provided with fine shallow punctures. Scutellum regularly triangular, feebly acute at apex.

Elytra relatively short, EL/EW 2.25–2.75 (M 2.46) in ♂, 2.08–2.90 (M 2.40) in ♀; sides with strongly rounded humeri, gently arcuate at a level between basal and apical fourth, then arcuately narrowed to apices which are slightly arcuate, without any spine at outer or inner angle; disc almost evenly convex, though distinctly depressed near suture in basal fifth, densely provided with fine shallow punctures.

Ventral surface almost smooth, provided with a few coarse punctures on prosternum, with densely minute punctures on meso- and metathoraces, and abdomen; anal ventrite in ♂, 2/5 the length of basal width, distinctly arcuate at apical margin.

Legs relatively short and slender; hind legs 1.4–1.6 times as long as elytra, with femur gradually swollen apicad, exceeding elytral apex at apical fifth, 1st tarsal segment 1.5–2.0 times as long as the following two segments combined.

Male genitalia. Median lobe almost 1/3 the length of elytra, slightly arcuate in profile; dorsal plate slightly wider than ventral plate in apical eighth, rounded at apex; ventral plate almost parallel-sided in basal 3/4 then strongly narrowed to apex which is sharply pointed at the extremity; median struts slender, almost half the length of median lobe. Endophallus largely sparsely provided with minute spinous spicules behind crescent-like sclerites, densely covered with minute crenulate spicules on apical fifth. Tegmen elongate, distinctly shorter than median lobe; parameres narrow, nearly 2/5 the length of tegmen, divided in apical third, with lobe moderately narrowed in weak arcuate line to apex, provided with numerous short setae and a few relatively long setae; basal ridge slightly raised; ring part almost approximate in apical 2/5. Eighth tergite elongate and quadrate, moderately narrowed from apical fourth to apex which is transversely truncate, provided with numerous short to long-sized setae. Eighth sternite distinctly narrower than 8th tergite, apical margin bi-arcuately rounded, triangularly concave near middle.

Female genitalia. Coxite lobe ovoid, provided with a few relatively long setae. Stylus elongate, almost 3/5 in length to coxite lobe, gently dilated apicad. Bursa copulatrix very large, almost circular, though strongly constricted in basal half. Spermatheca relatively narrow, moderately arcuate, bluntly pointed at apex; gland attached at apical third; duct thin, slightly sinuate in basal 2/3, coiled multiple times in apical third.

#### Type series.

Holotype ♂, Ronghua, Fuxing Township, alt. 500m, Taoyuan County, N. Taiwan, 9–III–1997, C.-C. Chen leg. Allotype ♀, Nanshansi, Ren’ai Township, Nantou County, C. Taiwan, 21–III–1979, T. Ito leg. Paratypes (7♂♂, 22♀♀): [Taoyuan County, N. Taiwan] 1♂, 1♀, Sule, Fuxing Township, 26–III–1979, T. Ito leg. [Hsinchu County, N. Taiwan] 1♂, 3♀♀, Shihlei, Jianshi Township, 6–III–2010, Y.-L. Lin leg. [Hualien County, E. C. Taiwan] 1♀, Bilyu, Xiulin Township, 2,200m, 29–III–1981, T. Shimomura leg. [Nantou County, C. Taiwan] 1♀, same locality and collector as the holotype, 17–III–1979; 2♂♂, 2♀♀, same locality, 19–III–1978, C.-K. Yu leg.; 1♀, same locality, 16–III–1978, J. Ito leg.; 1♀, same locality and collector, 17–III–1978; 1♂, 2♀♀, same locality and collector, 19–III–1978; 1♀, same locality and collector, 22–III–1978; 1♀, same locality and collector, 25–III–1978; 1♀, same locality and collector, 26–III–1978; 1♀, same locality, 26–III–1978, Y. Ito leg.; 1♀, same locality and collector, 31–III–1978; 1♀, same locality and collector, 28–III–1979; 1♀, same locality, 14–IV–1978, M. Ito leg.; 1♂, same locality, 21–III–1979, T. Ito leg.; 1♀, same locality, 11–IV–1985, J.-J. Luo leg.; 1♀, Tseifong, Ren’ai Township, 4–IV–1971, B.-S. Chang leg.; 1♀, Mt. Guandao Shan, Ren’ai Township, 14–IV–1973, J.-J. Luo leg. Holotype and allotype are preserved in NMNS, and paratypes are in EUMJ, HUM, MMNS, NHMO and the private collections of the above collectors.

#### Geographical distribution.

Taiwan.

#### Comments.

*Amamiclytus juni* sp. n. and the following new species, *Amamiclytus yulongi* sp. n. are both short with rounded body with a similar pattern of white pubescence on pronotum, elytra and ventral surface. They form an isolated species group among the Taiwanese members of the genus. These two species are also similar in regards to the male genitalia, for instance, in median lobe with long and shapely pointed ventral plate which is well exposed from ventral view, short and simply pointed lobes of parameres.

Most of the type series of *Amamiclytus juni* sp. n. were collected in early spring season in northern and central Taiwan nearly thirty years ago. According to our original field observations, this new species was not so rare and usually found on the blossoms of *Castanopsis* oak together with the other *Amamiclytus* species.

**Figures 65–70.**
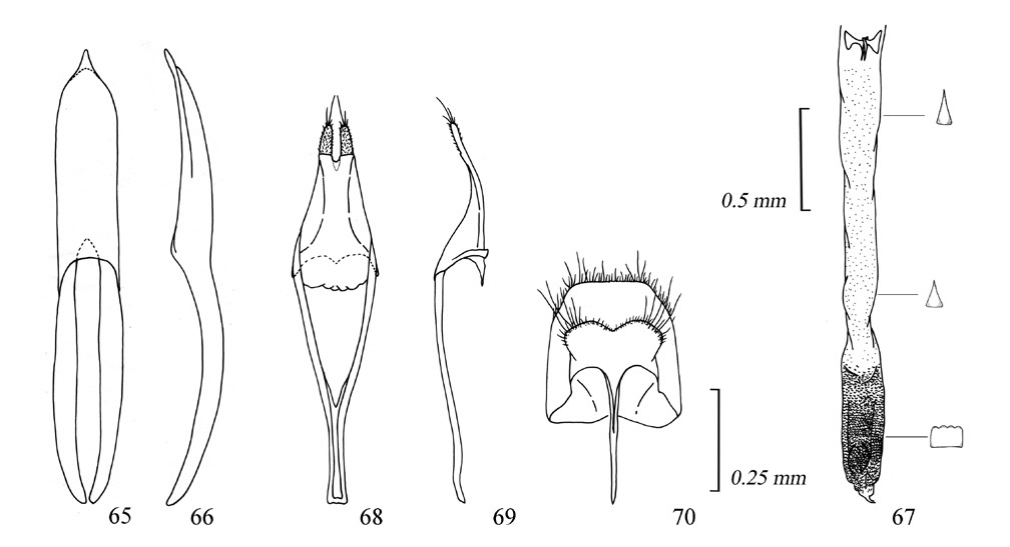
Male genitalia of *Amamiclytus juni* sp. n.: **65** Median lobe, ventral view **66** ditto, lateral view **67** endophallus **68** tegmen, ventral view **69** ditto, lateral view **70** abdominal segment 8, ventral view.

**Figures 71–76.**
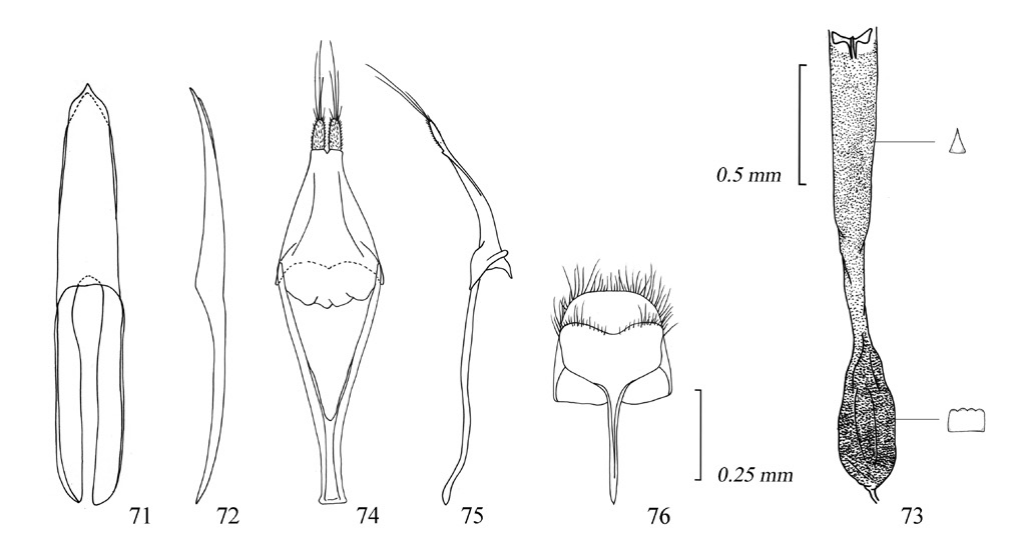
Male genitalia of *Amamiclytus yulongi* sp. n.: **71** Median lobe, ventral view **72** ditto, lateral view **73** endophallus **74** tegmen, ventral view **75** ditto, lateral view **76** abdominal segment 8, ventral view.

### Amamiclytus yulongi sp. n.

urn:lsid:zoobank.org:act:6FEFB8FD-B296-4BB1-BA4E-0DE85E94B814

http://species-id.net/wiki/Amamiclytus_yulongi

Figs 19–21 39 71–76 

#### Description.

Male and Female. Body length (from vertex to apices of elytra) 4.0–4.7 mm in ♂, 4.9–5.1 mm in ♀. Colour almost as in *Amamiclytus juni* sp. n., though more strongly glossy on elytra. Hairs and pubescence almost as in *Amamiclytus juni* sp. n.; head more densely clothed with pale gray pubescence on frons in both sexes; antennae densely with pale pubescence on segments 4–11; pronotum thinly with pale hairs, sparsely with pale gray pubescence; elytra densely with light brown pubescence throughout, *B* very sparse, *S* on basal tenth relatively small and oblong, *La* on basal third semicircular, almost always isolated, rarely arcuate and attaining to *S*, *Lp* on apical 2/5 more or less narrow, almost transverse, *A* not so narrow; *Msl* and *Mss* distinct, *Mta* moderately pubescent, not so dense; *V1* as in *Amamiclytus juni* sp. n., *V2* distinct, *V3* and *V4* sometimes disappeared.

Head almost as in *Amamiclytus juni* sp. n., though more densely finely punctured, with a thin feeble median line on frons; HW/PW 0.80–0.94 (M 0.88) in ♂, 0.71–0.95 (M 0.86) in ♀. Antennae as in *Amamiclytus juni* sp. n. Pronotum almost as in *Amamiclytus juni* sp. n.; disc gently convex throughout, especially raised near middle of basal margin, with punctation deeper and slightly large; PL/PW 1.06–1.11 (M 1.09) in ♂, 0.92–1.33 (M 1.08) in ♀, PB/PA 0.93–1.00 (M 0.95) in ♂, 0.80–0.94 (M 0.88) in ♀, PW/EW 0.80–0.90 (M 0.85) in ♂, EL/PL 2.75–3.22 (M 2.93) in ♂, 2.57–3.40 (M 3.04) in ♀, 0.70–0.85 (M 0.79) in ♀. Scutellum as in *Amamiclytus juni* sp. n. Elytra almost as in *Amamiclytus juni* sp. n.; sides gently arcuate at a level between basal eighths and apical fourth; disc with sparse but more or less deep punctation; EL/EW 2.48–2.90 (M 2.71) in ♂, 2.34–2.83 (M 2.54) in ♀. Ventral surface almost as in *Amamiclytus juni* sp. n., though more sparsely punctured on abdomen; anal ventrite in ♂ more or less triangularly produced at middle of apical margin. Legs almost as in *Amamiclytus juni* sp. n., though exceeding elytral apices at apical tenth.

Male genitalia. Basically similar to those of *Amamiclytus juni* sp. n., though median lobe more slender, with ventral plate not so strongly pointed apicad. Median lobe 1/3 the length of elytra, gently arcuate in profile; dorsal plate slightly wider than ventral plate in apical 3/5, distinctly narrowed to apex which is slightly pointed; ventral plate almost parallel-sided in basal 2/5 then gently narrowed to apex, and strongly narrowed to apical 1/12, which is sharply pointed at the extremity, shortly exposed from ventral view; median struts slender, almost half the length of median lobe. Endophallus densely covered with minute spinous spicules in apical fifth. Tegmen elongate, slightly shorter than median lobe; parameres relatively wide, nearly 2/5 the length of tegmen, divided in apical fifth, with lobe narrowed in gently arcuate line to apex, approximate and subparallel at inner margins, rather narrowly rounded at apex which is provided with numerous short and a few very long setae; basal ridge slightly raised; ring part almost approximate and parallel in apical third. Eighth tergite elongated and quadrate, gently narrowed from apical 3/4 to apex which is gently arcuate, provided with numerous long setae. Eighth sternite transverse, nearly equal in width to 8th tergite, apical margin arcuately oblique towards middle.

Female genitalia. Almost as in *Amamiclytus juni* sp. n., though bursa copulatrix smaller, semicircular in apical 2/5, moderately narrowed in basal 3/5.

#### Type series.

Holotype ♂, Dalu Forest Road, Wufeng Township, alt. 1,400~1,100m, Hsinchu County, N. Taiwan, 27–IV–2008, Y.-L. Lin leg. Allotype ♀, same data as the holotype. Paratypes (3♂♂, 10♀♀): [Taoyuan County, N. Taiwan] 1♀, Ronghua, Fuxing Township, 7–IV–1971, B.-S. Chang leg.; 1♀, Sihleng, Fuxing Township, 19–III–2000, 1♀, Y.-L. Lin leg. [Hsinchu County, N. Taiwan] 1♀, same locality and collector as the holotype, 19–IV–2008. [Yilan County, N. Taiwan] 1♀, Mingchih, Datong Township, 29–V–1978, J. Ito leg. [Nantou County, C. Taiwan] 1♂, Nanshansi, Ren’ai Township, 19–III–1978, C.-K. Yu leg.; 1♀, Mt. Guandao Shan, Ren’ai Township, alt. 1,500m, 5–V–1985, K. Kusama leg.; 1♂, Lianhuachih, Yuchi Township, 27–III–1980, K. Matsuda leg. [Hualien County, E. C. Taiwan] 1♀, Bilyu, Xiulin Township, 2,200m, 29–V–1978, Y. Oda leg. [Pingtung County, S. Taiwan] 1♀, Mt. Dahan Shan, Chunri Township, alt. 1,200m, 9–III–2006, Y.-L. Lin leg.; 1♂, same locality and collector, 1–III–2007; 1♀, same locality and collector, 9–III–2007; 1♀, Siaoguei Lake, Wutai Township, alt. 1,500m, 2–V–1998, C.-C. Chen leg.; 1♀, same locality and collector, 12~14–V–2008. Holotype and allotype are preserved in NMNS, and paratypes are in EUMJ, HUM, MMNS, NHMO and the private collections of the above collectors.

#### Geographical distribution.

Taiwan.

#### Comments.

*Amamiclytus yulongi* sp. n. is closely related in the external and genitalic morphology to *Amamiclytus juni* sp. n., but it can be distinguished from the latter by strongly glossy body, more coarsely punctured pronotum, sparse white pubescence on elytral bases, dense minute spinous spicules on the endophallus, gently arcuate apical margin of 8th tergite and not so strongly constricted basal part of bursa copratrix.

This new species is rather rare among the Taiwanese members of the genus, and sometimes found on the blossoms of *Castanopsis* and the other tree blossoms.

Four female paratypes show very peculiar variation in colour and pubescence as follows: 1) Body black with weak bluish tinge; 2) pronotum, elytra and ventral surface rather densely clothed with pale gray pubescence; 3) *S* and *La* completely joining; 3) *Lp* very wide, almost 4 times as wide as that of normal individuals, completely attaining both external and sutural margins; 4) *A* very wide, almost twice in width to that of normal individuals. These questionable specimens are completely consistent in both external and genitalic morphologies with the type series of *Amamiclytus  yulongi* sp. n. in spite of the peculiar external appearance.

**Figures 77–82.**
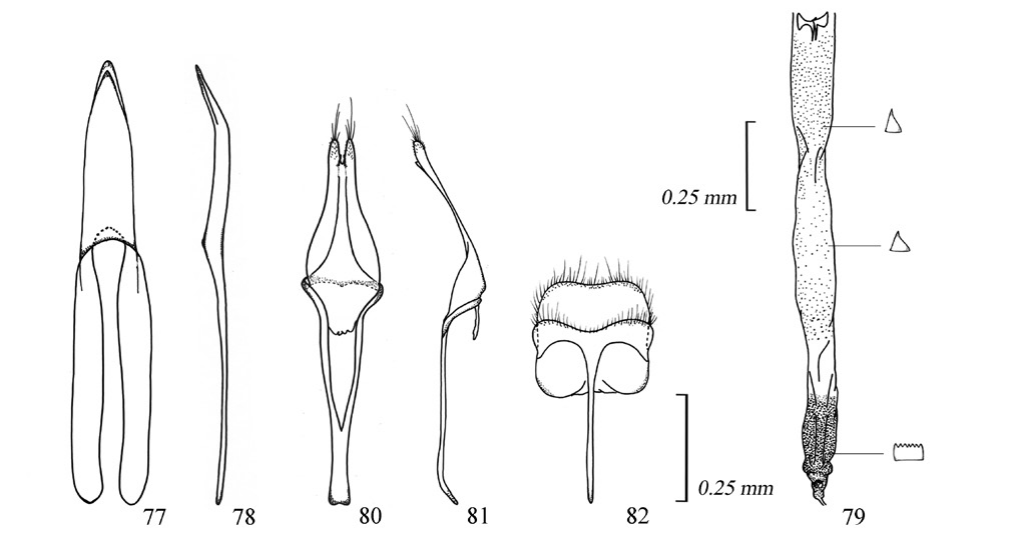
Male genitalia of *Amamiclytus hirtipes* (Matsushita), comb. n.: **77** Median lobe, ventral view **78** ditto, lateral view **79** endophallus **80** tegmen, ventral view **81** ditto, lateral view **82** abdominal segment 8, ventral view.

### Amamiclytus hirtipes

(Matsushita, 1940) comb. n.

http://species-id.net/wiki/Amamiclytus_hirtipes

Figs 4 22–23 24–25 40 77–82

Rhaphuma hirtipes Matsushita, 1940: 52; type locality: Formosa (Hori).

#### Description.

Male and Female. Body length (from vertex to apices of elytra) 4.0–5.8 mm in ♂, 4.4–6.0 mm in ♀. Large and slender species with more or less matted body, long antennae and legs. Colour black, relatively matted especially on elytra, more or less brownish on meso- and metathoraces, antennae and legs, yellowish brown on mouthparts except for black mandibles and anal ventrite. Body densely clothed with fine pale pubescence, sparsely with erect, long, pale hairs on undersides of fore femur, apical 2/5 of mid and hind femora, and all tibiae especially on hind pair; head thinly pubescent, scattered with a few erect, long, pale hairs, densely with pale gray pubescence on frons in ♂ and sparsely so in ♀; antennae with relatively long pale brown hairs along undersides of segments 3–5; pronotum thinly pubescent, largely exposing disc, without erect, pale hairs, rather sparsely with white pubescence at sides of basal margin, not formed conspicuous *Pb*; scutellum with a few pale pubescence; elytra rather densely with pale brown pubescence, without erect, pale hairs, *B* around scutellum relatively sparse, *S* on basal eighth small and longitudinally oblong, *La* on basal 3/10 semicircular, slightly oblique, *Lp* on apical 3/10 almost complete, narrow, weakly arcuate, *A* very narrow; prosternum sparsely with white pubescence near middle, *Msl* and *Mss* distinct, *Mta* rather sparse along posterior margin of metasternum, dense and entire on apical 2/3 of metepisternum; *V1* and *V2* at sides dense though narrowed to middle.

Head across eyes slightly narrower than pronotum, HW/PW 0.89–0.95 (M 0.93) in ♂, 0.85–0.95 (M 0.90) in ♀; frons as long as wide, arcuately dilated apicad, with a fine smooth median line, closely coarsely punctured; clypeus almost flattened; vertex raised towards antennal cavities which are separated from each other by 2/5 the width of occiput; occiput distinctly convex, closely reticulate. Antennae thin and long, not thickened apicad, attaining apical third in ♂ or half in ♀ of elytra, with scape almost cylindrical, 3rd segment 1.4–2.0 times as long as 4th segment, middle segments weakly thickened at apices, terminal segment distinctly elongate in ♂.

Pronotum usually distinctly longer than wide, rather weakly arcuate at sides; PL/PW 1.05–1.38 (M 1.23) in ♂, 1.00–1.40 (M 1.16) in ♀, PB/PA 0.92–0.94 (M 0.93) in ♂, 0.93–0.94 (M 0.93) in ♀, EL/PL 2.85–3.60 (M 3.18) in ♂, 2.77–3.73 (M 3.21) in ♀, PW/EW 0.71–0.90 (M 0.80) in ♂, 0.63–0.88 (M 0.76) in ♀; disc distinctly convex though depressed above, densely provided with uniform large shallow punctures. Scutellum regularly triangular, acute at apex.

Elytra long, slender, nearly or more than three times as long as wide, EL/EW 2.83–3.60 (M 3.10) in ♂, 2.50–3.05 (M 2.80) in ♀; sides with completely rounded humeri, gently arcuate at a level between basal fourth and apical 2/5 then arcuately narrowed to apices which are obliquely arcuate with blunt teeth at external angles; disc almost evenly convex, closely provided with fine shallow punctures.

Ventral surface almost smooth, provided with a few coarse punctures on prosternum, minute ones on meso- and metathoraces, and abdomen; ♂ anal ventrite 7/10 the length of basal width, weakly arcuate at apical margin.

Legs long and slender; hind legs 1.6–2.0 times as long as elytra, with femur gradually swollen apicad, slightly compressed, exceeding elytral apex at apical fifth, 1st tarsal segment 1.5–2.2 times as long as the following two segments combined.

Male genitalia. Median lobe almost 1/3 the length of elytra, in lateral view almost straight though distinctly bent ventrad in apical 2/5 of apical lobe; dorsal plate almost equal in width to or a little longer than ventral plate, gently narrowed to apex which is bluntly pointed; ventral plate almost parallel-sided in basal 3/4 then gently narrowed to apex which is more or less sharply pointed at the extremity; median struts slender, almost half the length of median lobe. Endophallus sparsely provided with minute spinous spicules behind crescent-like sclerites from basal 1/3 to 7/10, densely covered with minute serrate sclerites in apical fifth. Tegmen elongate, slightly shorter than median lobe; parameres elongate, nearly 2/5 the length of tegmen, divided in apical fifth, with lobe almost parallel-sided, moderately convergent apicad, slightly oblique at inner margins, almost rounded at apices which are provided with numerous short and a few long setae; basal ridge slightly raised; ring part parallel in apical third. Eighth tergite quadrate, arcuate at sides of apical margin which is slightly emarginate, provided with numerous short and long setae. Eighth sternite strongly transverse, rather weakly emarginate at apical margin.

Female genitalia. Coxite lobe ovoid, scattered with a few long and short setae. Stylus almost half in length to coxite lobe, elongate, moderately dilated apicad. Bursa copulatrix more or less large, weakly narrowed in basal half. Spermatheca narrow and strongly arcuate in apical half; gland oblong, attached at apical half; duct thin, short, slightly sinuate, not coiled.

#### Specimens examined.

1♂ (holotype), Hori, Formosa, 20–II–1939, (HUM). [Taoyuan County, N. Taiwan] 1♂, 1♀, Syuanyuan, Fuxing Township, alt. 800m, 18–IX–2005, Y.-L. Lin leg.; 6♂♂, 8♀♀, same locality and collector, alt. 700m, 17–X–2009. [Hsinchu County, N. Taiwan] 1♂, Dalu Forest Road, Wufeng Township, alt. 1,100~1,400m, 13–VII–2003, Y.-L. Lin leg. [Nantou County, C. Taiwan] 2♂♂, Mt. Lushan, Ren’ai Township, 23–VIII–1987, J.-J. Luo leg.; 10♂♂, 6♀♀, Gaofeng, Ren’ai Township, 19–IX–2006, native collector leg.; 3♂♂, Mt. Hewang, Ren’ai Township, alt. 1,650m, 9–IX–2009, C.-C. Chen leg. [Kaohsiung County, S. Taiwan] 8♂♂, 9♀♀, near Liouguei Township, 5–X–1983, W.-L. Chen leg.; 2♂♂, same locality, 5–X–1984, B.-R. Chin leg.; 15♂♂, 16♀♀, Douna, Maolin Township, 8–IX–2007, W.-S. Lin leg. [Pingtung County, S. Taiwan] 5♂♂, 4♀♀, Mt. Dahan Shan, Chunri Township, alt. 1,200m, 12–X–2003, Y.-L. Lin leg. 1♂, Mt. Dahan Shan, alt. 1,300m, 25–X–2007, W.-S. Lin leg.

#### Geographical distribution.

Taiwan.

#### Comments.

This clytine beetle is formally treated here as a member of the genus *Amamiclytus* Ohbayashi. As was written in “Historical review” on the earlier page, this species was first described under the genus *Rhaphuma* Pascoe (Matsushita 1940), and later was transferred to *Amamiclytus* by misidentification (Kojima et al. 1965). The previous authors, such as Kojima et al. (1965), had never examined the holotype of *Rhaphuma hirtipes*, and confused it as the senior synonym of *Amamiclytus nobuoi* Ohbayashi. We carefully examined the holotype specimen of *Rhaphuma hirtipes* deposited in the Hokkaido University Museum, Sapporo, and recognized it as actually a member of the genus *Amamiclytus*.

*Amamiclytus hirtipes* (Matsushita, 1940), comb. n. is easily distinguished in external appearance from the other Taiwanese members of the genus. This species has a more elongate body on average, with the elytra nearly or more than three times as long as wide, and the very long slender antennae attaining the apical third of elytra in male or apical half in female, with less thickened middle segments and a distinctly elongate terminal segment, especially in male. From the sparse arrangement of white pubescence near base of pronotum, this species is easily separable from several similar species which has the distinct white pubescent maculation so called “*Pb*”. Concerning the morphology of male genitalia, this species is basically similar to those of *Amamiclytus nobuoi* Ohbayashi, *Amamiclytus subnitidus* Holzschuh and *Amamiclytus setiger* sp. n. in spite of its unique external morphology.

It is very interesting that the adults of *Amamiclytus hirtipes* mainly appear in the autumn season in September and October, and have never been found in springtime as is the case with all of the other members of the genus. The species is not so rare in this season, and usually found on tree blossoms. However two exceptional records are known; one male collected in mid July from Dalu Forest Road in northern Taiwan; the holotype male collected in February in central Taiwan.

## Discussion

In spite of its uniqueness, *Amamiclytus* Ohbayashi shows relationship in external morphology with the genera *Rhaphuma* Pascoe, *Chlorophorus* Chevrolat and *Demonax* Thomson as follows: 1) body including legs and antennae usually elongate; 2) antennae thin and long, almost exceeding apical halves of elytra in male, with segment 3 longer than scape; 3) eyes large and approximate to each other; 4) genae relatively shallow in frontal view; 5) mandible along inner margin smooth; 5) labial and maxillary palpi has a distinct sexual dimorphism in each terminal segment.

Han and Niisato (2009) suggested that the structure of endophallus in male genitalia is one of the important characters to distinguish the genera of the tribe Clytini. *Amamiclytus* is clearly defined by this character and distinguishable from the other genera of the tribe, though it has a close relationship with the genera *Rhaphuma*, *Chlorophorus* and *Demonax*. These three related genera show the following features on the spicules of their endophallus; 1) *Rhaphuma*: usually without any spicules near base of endophallus, or weakly, rather sparsely provided with several kinds of spicules on the whole of endophallus; 2) *Chlorophorus*: densely provided with large-sized sclerotized spicules on the whole of endophallus; 3) *Demonax*: basically similar to those of *Amamiclytus*, though has a pair of sclerotized lines consisting of minute or medium-sized spinous spicules on the apical part of endophallus.

A total of seven species of *Amamiclytus* from Taiwan and the Ryukyu Islands of Southwest Japan are provisionally divided into four morphological groups mainly based on both the male and female genitalia as follows.

Group I: *Amamiclytus subnitidus* Holzschuh, 1984, *Amamiclytus setiger* sp. n. and *Amamiclytus nobuoi  nobuoi* Ohbayashi, 1964, *Amamiclytus nobuoi akusekianus* Niisato, 2005.

Group II: *Amamiclytus nubilus* sp. n.

Group III: *Amamiclytus juni* sp. n. and *Amamiclytus yulongi* sp. n.

Group IV: *Amamiclytus hirtipes* (Matsushita, 1940), comb. n.

Group I is recognized by small, slightly elongate, glossy (usually strongly) body with sparsely or thinly pubescent elytra, and is provisionally composed of three species. The members of this group share the following features in male genitalia:- the long median struts which are 3/5 to 7/10 the length of median lobe, as well as the long parameres which are half the length of tegmen. In female genitalia, they share the distinctly short coxite lobes which are almost equal to the length of stylus, and strongly wholly coiled spermathecal duct in the whole length. *Amamiclytus nobuoi* is provisionally placed in this group and basically identical in the above characters with *Amamiclytus subnitidus* and *Amamiclytus setiger*. However, the male genitalia are rather distinctly shortened and similar to those of *Amamiclytus hirtipes* (Group IV), *Amamiclytus juni* and *Amamiclytus yulongi* (Group III). Besides, *Amamiclytus nobuoi* is provided with a sutural spot on elytra behind scutellum (“*S*”) which is absent in *Amamiclytus subnitidus* and *Amamiclytus setiger*.

Group II is composed of a single species, *Amamiclytus nubilus*, characterized by matted, rather elongate body with dense pubescence on the elytra, and somewhat similar in general appearance to *Amamiclytus hirtipes* except for the presence of a distinct basal band on pronotum (“*Pb*”) and the absence of basal bands near elytral bases (“*B*”). Spermatheca of female genitalia is strongly arcuate, with simple, not coiled duct. Also, the male genitalia are basically similar in the proportion of median struts and parameres to the Group I, though the median lobe is not so elongate and the ring part of tegmen is not expanded apicad as in those of Group I.

Group III composed of *Amamiclytus juni* and *Amamiclytus yulongi*, is characterized by short, broadened and matted body, rather transverse pronotum with distinctly arcuate sides, without white pubescence near the basal margin. The independence of this group is strongly supported by the structures of both male and female genitalia. In the female genitalia, the coxite lobes are long, about twice the length of stylus, spermathecal duct is strongly coiled in apical third, and bursa copulatrix is very large. It has the shortened male genitalia, of which the median struts are half the length of median lobe, and the parameres are 2/5 the length of tegmen.

Group IV is also monotypic at least in the present sense, composed of a single species, *Amamiclytus hirtipes*, and is clearly distinguished from the other groups of the genus by very large and slender body on average, with sparse “*Pb*” on the pronotum and without “*B*” on the elytra. However, *Amamiclytus hirtipes* has close relationship in female genitalia with *Amamiclytus nubilus*; spermatheca is strongly curved near middle, with gland attached at the middle of spermatheca, and the duct is not coiled, only weakly sinuate throughout. Male genitalia of *Amamiclytus hirtipes* are also similar to those of *Amamiclytus nobuoi* (Group I), and *Amamiclytus juni* and *Amamiclytus yulongi* (Group III).

**Figure 83.**
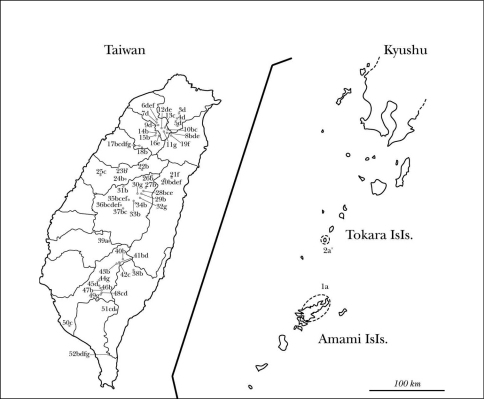
Distribution of species and collecting sites: **a** *Amamiclytus nobuoi nobuoi* Ohbayashi **a’** *Amamiclytus nobuoi akusekianus* Niisato **b** *Amamiclytus subnitidus* Holzschuh **c** *Amamiclytus setiger* sp. n. **d** *Amamiclytus nubilus* sp. n. **e** *Amamiclytus juni* sp. n. **f** *Amamiclytus yulongi* **g** *Amamiclytus hirtipes* (Matsushita). [N. Ryukyus, Kagoshima Pref., Japan] **1** Amami-Ôshima Is., **2** Akuseki-jima Is., Tokara Isls. [Taipei County, N. Taiwan] **3** Wulai Township, **4** Sinsian, Wulai Township, **5** Fushan, Wulai Township [Taoyuan, County, N. Taiwan] **6** Ronghua, Fuxing Township, **7** Baling, Fuxing Township, **8** Sihleng, Fuxing Township, **9** Syuanyuan, Fuxing Township, **10** near Mt. Lala Shan, Fuxing Township, **11** Gaoyi, Fuxing Township, **12** Sule, Fuxing Township, **13** Shangbaling, Fuxing Township [Hsinchu County, N. Taiwan] **14** Mt. Lidong Shan, Jianshi Township, **15** Yufong, Jianshi Township, **16** Shihlei, Jianshi, Township, **17** Dalu Forest Rd., Wufeng Township, **18** Dalu Forest Rd., East Feeder, Wufeng Township [Yilan County, N. Taiwan] **19** Mingchih (new name), Datong Township [Hualien County, E. C. Taiwan] **20** Bilyu, Xiulin Township, **21** Sinbaiyuan [Taichung County, C. Taiwan] **22** Deji Reservoir, Heping Township, **23** Mt. Anma Shan, Heping Township, **24** Lilengsi Forest Rd., Heping Township, **25** Mt. DaKeng, Beitun District, Taichung City, [Nantou County, C. Taiwan] 26, Bilyusi, Ren’ai Township, **27** Meifong, Ren’ai Township, **28** Cueifong, Ren’ai Township, **29** Songgang~Meifong, Ren’ai Township, **30** Mt. Hewang, Ren’ai Township, **31** Meiyuan, Ren’ai Township, **32** Lushan, Ren’ai Township, **33** Nanshansi, Ren’ai Township, **34** Gaofeng, Ren’ai Township, **35** Mt. Guandao Shan, Ren’ai Township, **36** Lianhuachih, Yuchi Township, **37** Sun Moon Lake, Yuchi Township [Taitung County, C. Taiwan] **38** Siangyang~Liyuan [Chiayi County, S. Taiwan] **39** Jhaoping, Alishan Township [Kaohsiung County, S. Taiwan] **40** Chuyushan Taoyuan Township, **41** Tengjhih, Taoyuan Township, **42** Mt. Sinan, **43** Mt. Pao Shan, **44** near Liouguei Township, **45** Wetuan, Liugui Township, **46** Mt. Shanping, **47** Mt. Nanfenshan, Liouguei Township, **48** Shanping, Maolin Township, **49** Douna, Maolin Township, **50** Fengshan, [Pingtung County. S. Taiwan] **51** Siaoguei Lake, Wutai Township, **52** Mt. Dahan Shan, Chunri Township.

## Supplementary Material

XML Treatment for Amamiclytus

XML Treatment for Amamiclytus nobuoi nobuoi

XML Treatment for Amamiclytus nobuoi akusekianus

XML Treatment for Amamiclytus subnitidus

XML Treatment for Amamiclytus setiger

XML Treatment for Amamiclytus nubilus

XML Treatment for Amamiclytus juni

XML Treatment for Amamiclytus yulongi

XML Treatment for Amamiclytus hirtipes
